# Highly efficient clustering of long-read transcriptomic data with GeLuster

**DOI:** 10.1093/bioinformatics/btae059

**Published:** 2024-02-03

**Authors:** Junchi Ma, Xiaoyu Zhao, Enfeng Qi, Renmin Han, Ting Yu, Guojun Li

**Affiliations:** Research Center for Mathematics and Interdisciplinary Sciences (Frontiers Science Center for Nonlinear Expectations), Shandong University, Qingdao 266237, China; School of Mathematics, Shandong University, Jinan, Shandong 250100, China; School of Mathematics, Shandong University, Jinan, Shandong 250100, China; School of Mathematics and Statistics, Guangxi Normal University, Guilin 541000, China; Research Center for Mathematics and Interdisciplinary Sciences (Frontiers Science Center for Nonlinear Expectations), Shandong University, Qingdao 266237, China; Research Center for Mathematics and Interdisciplinary Sciences (Frontiers Science Center for Nonlinear Expectations), Shandong University, Qingdao 266237, China; Research Center for Mathematics and Interdisciplinary Sciences (Frontiers Science Center for Nonlinear Expectations), Shandong University, Qingdao 266237, China

## Abstract

**Motivation:**

The advancement of long-read RNA sequencing technologies leads to a bright future for transcriptome analysis, in which clustering long reads according to their gene family of origin is of great importance. However, existing de novo clustering algorithms require plenty of computing resources.

**Results:**

We developed a new algorithm GeLuster for clustering long RNA-seq reads. Based on our tests on one simulated dataset and nine real datasets, GeLuster exhibited superior performance. On the tested Nanopore datasets it ran 2.9–17.5 times as fast as the second-fastest method with less than one-seventh of memory consumption, while achieving higher clustering accuracy. And on the PacBio data, GeLuster also had a similar performance. It sets the stage for large-scale transcriptome study in future.

**Availability and implementation:**

GeLuster is freely available at https://github.com/yutingsdu/GeLuster.

## 1 Introduction

Long-read sequencing, which was originally slow, error-prone and costly, has seen remarkable progress over the past decade (Kovaka *et al.* 2023). And, long-read transcriptome sequencing has demonstrated its importance in exploring the intricate isoform landscapes in a wide variety of species including human (Byrne *et al.* 2017, Tseng *et al.* 2017, Nattestad *et al.* 2018, Sahlin *et al.* 2018), animals (Kuo *et al.* 2017, Walter and Puniamoorthy 2022, Marta *et al.* 2023), plants (Hoang *et al.* 2017), and etc. Long-read techniques are equipped with the ability to produce reads longer than 10 kb (Pollard *et al.* 2018, Amarasinghe *et al.* 2020), which are capable of spanning the full range of most eukaryotic transcripts, thus making it a powerful tool for studying transcriptome at the isoform level. However, current long-read sequencing platforms, including Pacific Biosciences (PacBio) and Oxford Nanopores Technologies (ONT), suffer from a high sequencing error rate (Weirather *et al.* 2017, Rang *et al.* 2018). Although both platforms developed strategies to improve the sequencing quality, such as the circular consensus sequencing (CCS) protocol of PacBio (Wenger *et al.* 2019) and the rolling circle amplification to concatemeric consensus (R2C2) method of ONT (Volden *et al.* 2018), the sequencing throughput is heavily decreased and are prone to systematic biases (Weirather *et al.* 2017, Gao *et al.* 2023). Consequently, ONT 1D cDNA and direct RNA-seq (Wang *et al.* 2021) are arguably the most cost-efficient of the long-read RNA-seq strategies (Gao *et al.* 2023).

For species where a high-quality reference is not available or for complex gene families, de novo clustering RNA-seq reads at the gene level are commonly required (Marchet *et al.* 2019, Sahlin and Medvedev 2020, Sahlin and Medvedev 2021). Clustering according to gene (rather than isoform) of origin retains more complete exon coverage for reads, and helps the downstream error correction process to be able to leverage exons that are shared between different splice isoforms (Sahlin and Medvedev 2020, Sahlin and Medvedev 2021).

Various computational methods have been developed specifically for clustering long-read RNA-seq data at the gene level in recent years. CARNAC-LR (Marchet *et al.* 2019) performed the clustering using the similarity graph that was built by pairwise alignments between sequencing reads, but it performed relatively worse especially for large datasets (Sahlin and Medvedev 2020). isONclust used a greedy, and quality value-based clustering strategy (Sahlin and Medvedev 2020). The clustering algorithm of RATTLE was also a greedy strategy, it performed a greedy deterministic clustering using a two-step k-mer based similarity measure to build the read clusters (de la Rubia *et al.* 2022). Nonetheless, when the dataset comprises only millions of reads, the running time of these tools can reach dozens or even hundreds of hours with dozens of GB of memory usage, which significantly limit their application in processing large-scale datasets. Highly efficient clustering methods are needed to achieve the maximum possible benefits of long-read RNA-seq technology in this setting.

In this research, we present GeLuster that is a highly efficient algorithm for clustering long-read transcriptome data according to their gene family of origin. Different from the traditional time-consuming all-vs-all pairwise alignment strategy or greedy strategy that deeply based on some kind of scoring system, GeLuster uses an innovative methodology. As is known, when a high-quality reference genome is available, gene cluster for the sequencing reads can be obtained via the alignments between the reads and the reference genome. Inspired by this fact, GeLuster first extracts the so-called pseudo reference from the raw sequencing reads, which expectantly correspond to the expressed genes for the sequencing data. Then, based on a global optimal alignment between the raw reads and the pseudo reference, GeLuster generate the pre-clusters. Since each sequence in the pseudo reference corresponds to a gene, the alignments intuitively cluster the sequencing reads into gene family. Then, by iteratively performing the above implementation, GeLuster efficiently get the final read clusters. Tested on nine real datasets and one simulated dataset, GeLuster exhibited higher clustering accuracy than the published tools. Moreover, GeLuster demonstrated significantly higher computational efficiency, where on the Nanopore data it ran 2.9–147 times as fast as the alternatives with only less than one-seventh of memory consumption, and two times faster with around one-fifth of memory usage on the PacBio data.

## 2 Materials and methods

### 2.1 GeLuster overview

**Figure 1.**
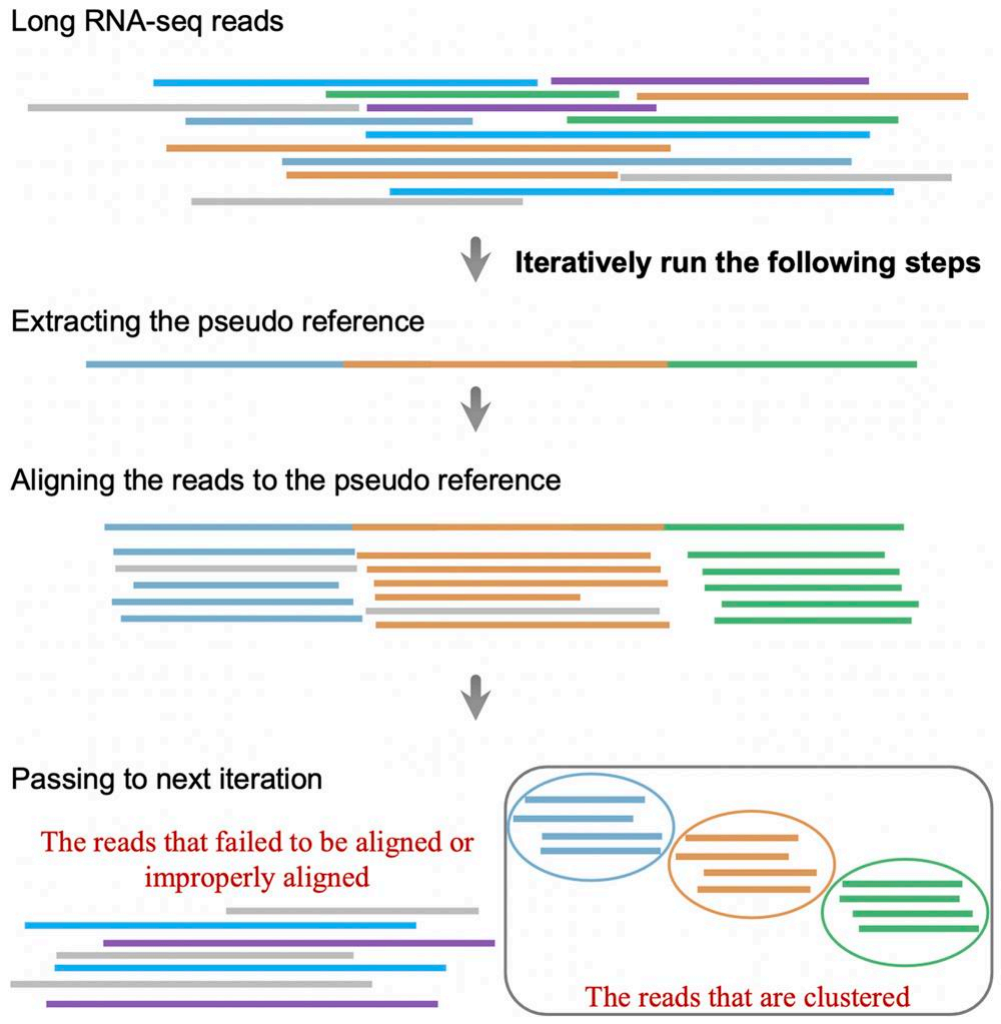
Flowchart of GeLuster.

In contrast to the traditional clustering strategies for the long- read RNA-seq data, such as clustering based on all-vs-all pairwise alignments between sequences, or greedy clustering according to some kind of scoring system between sequences, GeLuster uses a new methodology (Fig. 1).

As known, RNA-seq reads originate from gene expression, if the reference sequences of the expressed genes are available, clustering turns to be simple and intuitive with the implementation of aligning reads to the reference. Therefore, the basic idea behind GeLuster is to extract the so-called pseudo reference sequences from the raw sequencing reads, each of which is expected to correspond to an individual gene sequence. And, the pseudo reference will be taken as a good proxy of the potential genes. The sequencing reads are then aligned against the pseudo reference. Based on the alignments, reads from the same gene family will be naturally clustered together.

### 2.2 The GeLuster algorithm

The GeLuster package’s input is a FASTQ or FASTA file of long reads. The output for the clustering results is a text file with one line per read, which specifies the name of the read and the cluster to which the read belongs. The name of each read is represented by its index in the original FASTQ or FASTA file. Besides, each cluster is converted to be a FASTQ (or FASTA) file where each read’s sequence is retrieved from the original file. (See https://github.com/yutingsdu/GeLuster for more details). GeLuster runs in an iterative way, and it implements the following steps within each iteration.

*Extract the pseudo reference sequences from reads that need to be clustered*. The pseudo reference is actually a subset of the sequencing reads, which is projected to contain as much potential genes as possible for the dataset, meanwhile, a gene is expected to correspond to only one subsequence in the pseudo reference. As known, a very common situation is that a single gene can be transcribed into several kinds of transcripts due to alternative splicing. However, the transcripts from the same gene generally share a large amount of sequence(exons) (LeGault and Dewey 2013, Peng *et al.* 2013, Zhang and Wang 2014), accordingly, for a gene, we find a sequence that acts as a proxy of the gene.

Concretely, let R be the set of the reads that need to be clustered, we are trying to find a subset of R as the pseudo reference, denoted by P, such that P should contain enough sequencing information of the potential genes, while the similarity between each pair of sequences in P is as low as possible. In GeLuster algorithm, the similarity between two sequences is defined according to the number of the shared k-mers in both sequences. Consequently, the problem can be formulated into the following programming problem,

max |P|s.t.P⊆Rsimilaritys1,s2<α, for s1,s2∈P

where |P| denotes the number of sequences in P; s$\mathrm{imilarity}\left(s_{1},s_{2}\right)$ denotes the similarity between *s_1_* and *s_2_*, defined as the number of shared *k-mers* divided by the minimum number of *k-mers* in either sequence; α is a parameter, indicating the maximum degree of similarity between two different genes that we can tolerate.

However, long-read data generally suffers from high sequencing error rates. Thus, to improve computation efficiency and decrease memory usage, we use *minimizer* rather than *k-mer* in the above programming, where *minimizer* or (*w, k*)*-minimizer* of a sequence is the *k-mer* with smallest hash value in a surrounding window of *w* consecutive *k-mers* (Li 2016), which has been used in various ways in many bioinformatics programs (Li 2016, 2018, Marçais *et al.* 2019, Rowe 2019, Zheng *et al.* 2020).

Afterwards, for efficient calculation, we heuristically solve the programming problem using the following steps: (i) sort the reads in descending order by their length to process them one at a time in that order, denoted by $R=\{r_{1},\;r_{2},\;\ldots \}$; and (ii) set $P\;=\;\{r_{1}\}$, for each $r_{i}\in R$ and $r_{i}$ is longer than *L*, if $\mathrm{similarity}\left(r_{i},r_{j}\right)<\alpha$ for each $r_{j}\in P$, then let $P\;=\;P\cup \{r_{i}\}$. It is worth noting that in the above steps, a parameter *L* is introduced to constrain the length of the read that will be selected into the reference in the current iteration, which ensures the efficiency of the calculation to a certain extent. Moreover, *L* is not set to be fixed within different iterations, and the setting of *L* will be described in the following sections. As for the parameter α, it is obtained by training from our tests, and it is set to 0.03 by default in the current version of the GeLuster package.

*Align the sequencing reads to the pseudo reference to get the pre-clusters*. As mentioned above, the sequences in the pseudo reference theoretically correspond to the potential genes. Therefore, with the implementation of aligning the sequencing reads to the pseudo reference, reads will be clustered naturally. The alignment in the GeLuster package is performed by minimap2 (Li 2018) with the option: -ax splice -k 11. Based on the alignment in the SAM format, an alignment for a read is considered as a proper alignment if it satisfies the following conditions: (i) The alignment should be the primary alignment reported by minimap2. (ii) The number of matched bases for the alignment should be respectively greater than the number of mismatches, inserts, deletions, and soft clipped bases, however, if there is no secondary alignment for a read, the number of matches is allowed to be less than that of the soft clipped bases. After selecting the proper alignment of a read to the pseudo reference, the read clusters are produced naturally. Furthermore, it is worth mentioning that minimap2 is quite fast in processing long read alignment, and this is an important guarantee for the highly efficient of GeLuster. Afterward, for the reads that failed to align to the pseudo reference or failed to get a proper alignment, GeLuster will pass them as the input to the next iteration.

*Run the process iteratively*. As mentioned above, only the reads that are longer than a parameter L are considered to be chosen as a pseudo reference sequence in each iteration for the purpose of efficient calculation. Then, after the implementation of aligning, the reads failed to align or improperly aligned will be passed as the input to the next iteration. The number of iterations in the current version of GeLuster package has been set to be a user-friendly parameter, and GeLuster performs three iterations by default. Moreover, in order to adapt GeLuster to different types of long-read datasets, the parameter L is set dynamically. In the first iteration, it is set to be the N60 length of the reads that need to be clustered, while in the second iteration it is the N80 length, and in the third and the subsequent iterations it is the N90 length. The above configuration not only ensures the efficiency of GeLuster, but also ensures that the final clusters contain as much sequencing reads as possible based on our experiments and observation.

## 3 Results

To illustrate the ability of GeLuster in clustering long-read RNA-seq data, we compared it with two popular clustering tools isONclust (Sahlin and Medvedev 2020) and RATTLE (de la Rubia *et al.* 2022), both of which are specifically designed for long read transcriptome data. Besides, we tried to include another clustering tool CARNAC-LR (Marchet *et al.* 2019) in the comparison, however, it failed to produce the clustering results after having run over two months on any of our tested dataset. Thus, we were not able to compare with CARNAC-LR. We evaluated the performance of the clustering tools using both simulated and real datasets on various of accuracy metrics. Moreover, we investigated the comparison of the consumption of computing resources.

### 3.1 Datasets

In this research, one simulated dataset and nine real datasets, where the real datasets contained six ONT complementary DNA datasets, two ONT direct RNA datasets, and one PacBio dataset, were used to benchmark the clustering tools. The simulated dataset was generated by using realistic gene expression profiles with Trans-NanoSim (Hafezqorani *et al.* 2020). The scripts we used for simulating the dataset were similar to the previous study (Prjibelski *et al.* 2023), and the simulated dataset contains 1.5 million reads. Simulated data were generally generated by using a mathematical model and would not be able to capture the entire features of real biological data. For this reason, the performance evaluation based on real RNA-seq datasets is of great necessity. Here, nine real datasets were used for benchmarking. The six cDNA datasets were R1, the human brain sample with ∼3.64 million reads; R2, the human heart sample with ∼3.64 million reads; R3, the GM12878 cell line sample with ∼3.51 million reads; R4, the HGC-27 cell sample with ∼2.37 million reads; R5, the K562 cell sample with ∼1.71 million reads; and R6, another K562 cell sample with ∼4.43 million reads. And the two ONT dRNA datasets were R7 and R8, which were two HEK-293T cell samples with ∼1.82 and ∼1.90 million reads respectively. We also included a PacBio dataset R9, the hFOB cell sample with ∼1.66 million reads. The accession code of each dataset can be found in the Supplementary Notes.

### 3.2 Ground truth and evaluation metrics

We can explicitly know the true classes of the reads in the simulated dataset. However, for the real datasets, the ground truth of the sequencing reads remains unknown. Therefore, as did in previous study (Sahlin and Medvedev 2020), we aligned the sequencing reads to the main chromosome of the reference genome (version Human GRCh38) using minimap2 (the running command can be found in the Supplementary Notes). And, for each read, the primary alignment according to the output of minimap2 were selected. Afterward, the sequencing reads were clustered based on the mapped genomic positions. The reads that were overlapped with each other were clustered together, and we refer to the clusters of the reads based on the alignments as the ground truth of the sequencing reads. As for the reads that failed to be aligned, in the subsequent accuracy evaluations, we exclude them since they could not be assigned to a class. The detailed description for the constructed ground truth were listed in Supplementary Table S1. Although, it is possible to produce systemic misalignments due to bias of the aligner, or chimeric reads, it is generally safe to assume that a tool is more sensitive if its clustering results are more consistent with such a ground truth (Sahlin and Medvedev 2020).

We mainly used four accuracy metrics to evaluate the performance of the clustering tools, which were ARI, V-Measure, FMI, and NMI (the detailed definitions of these metrics could be found in the Supplementary Notes). To put it simply, the higher the four metrics, the better the clustering, and a perfect clustering achieves 1 on all these four metrics, while a random cluster assignment achieves these four metrics of 0.

### 3.3 Comparison against other tools

Based on the clustering results generated by the three tools GeLuster, RATTLE, and isONclust on the simulated and real datasets, we evaluated their accuracy performance in terms of the four aforementioned metrics, and evaluated their performance in the consumption of the computing resources. The running commands of the tools can be found in Supplementary Notes.

#### 3.3.1 Basic accuracy evaluation

**Figure 2.**
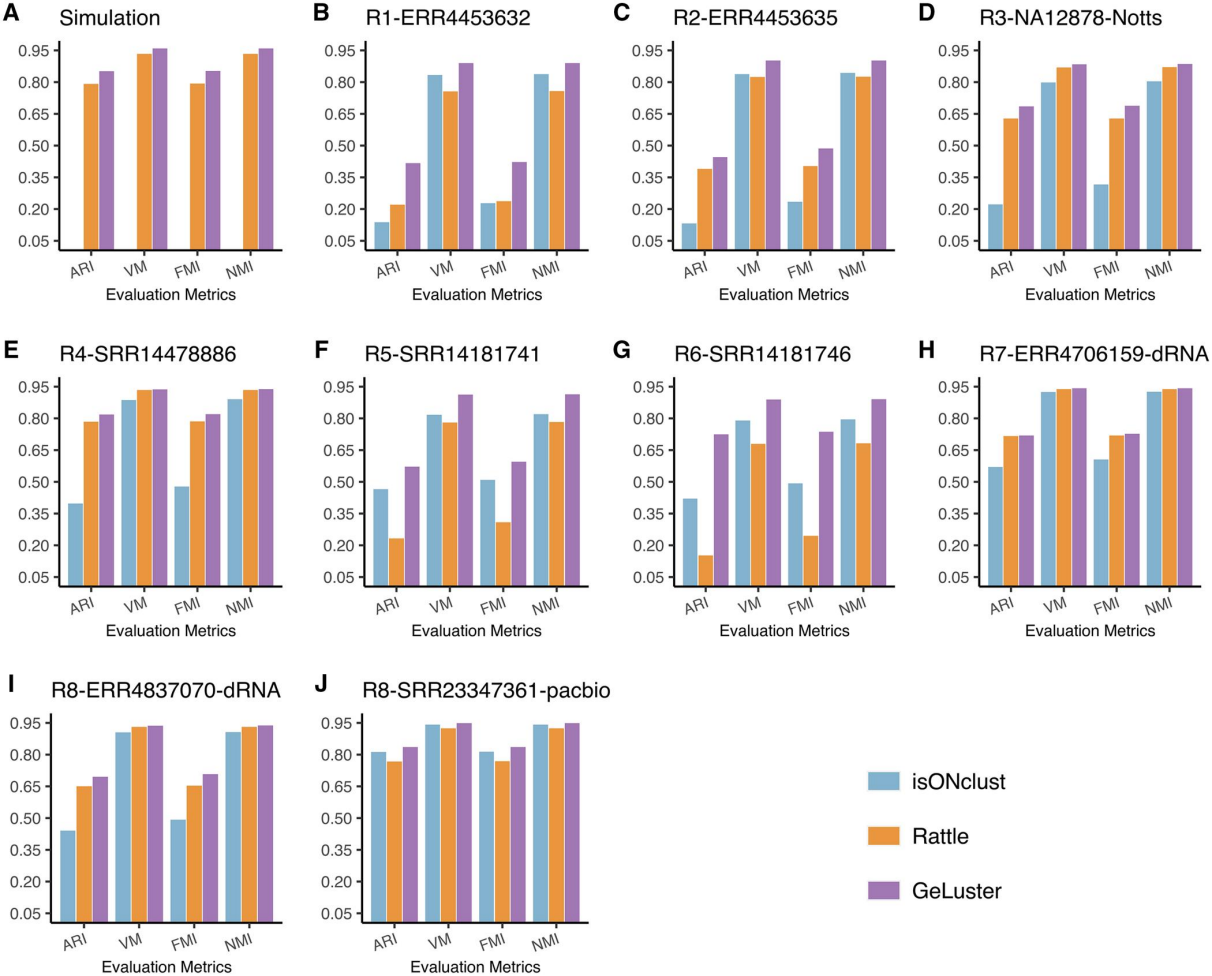
Basic accuracy evaluation (in terms of ARI, V-Measure, FMI, and NMI) of the clustering tools on the tested datasets. (A) corresponds to the simulated dataset, (B–G) correspond to the six cDNA datasets, (H) and (I) correspond to the two dRNA datasets, and (J) corresponds to the PacBio dataset.

In terms of the four basic accuracy metrics ARI, V-Measure, FMI, and NMI, GeLuster showed improved performance over the others on both the simulated and real datasets. Specifically, on the simulated dataset, GeLuster achieved an ARI score of 0.8521, a V-Measure score of 0.9590, an FMI score of 0.8533, and an NMI of 0.9590, while these four metrics for RATTLE were 0.7912, 0.9330, 0.7933, and 0.9330 (see Fig. 2A and Supplementary Table S2 for more details). Besides, it should be noted that isONclust failed to generate the clustering results after has run over one months on the simulated dataset. Thus, we did not include the comparison with it. On the six real ONT cDNA datasets, the ARI score of GeLuster ranged from 0.4173 to 0.8178, with an average of 0.6104, versus 0.1526 to 0.7845 for RATTLE with an average of 0.4014, and 0.1317 to 0.4654 for isONclust with an average of 0.2961. It indicated that averaged on the six datasets, GeLuster showed an improvement of >52% over the alternatives based on the ARI score. For the V-Measure score, GeLuster manifested an increase of 11.7% over RATTLE, and 9.1% over isONclust averaged on the six datasets. And, the comparisons in terms of FMI and NMI also demonstrated that GeLuster performed better than the other two methods (see Fig. 2B–G and Supplementary Tables S3–S8 for more details).

Furthermore, similarly as it did on the cDNA datasets, GeLuster maintained its superiority over the others on the dRNA datasets and the PacBio dataset (see Fig. 2H–J and Supplementary Tables S9–S11 for more details). On these grounds, GeLuster exhibited superior performance among all the compared tools regardless of any accuracy metrics.

#### 3.3.2 Running time and memory usage of the clustering tools on the real datasets

**Figure 3.**
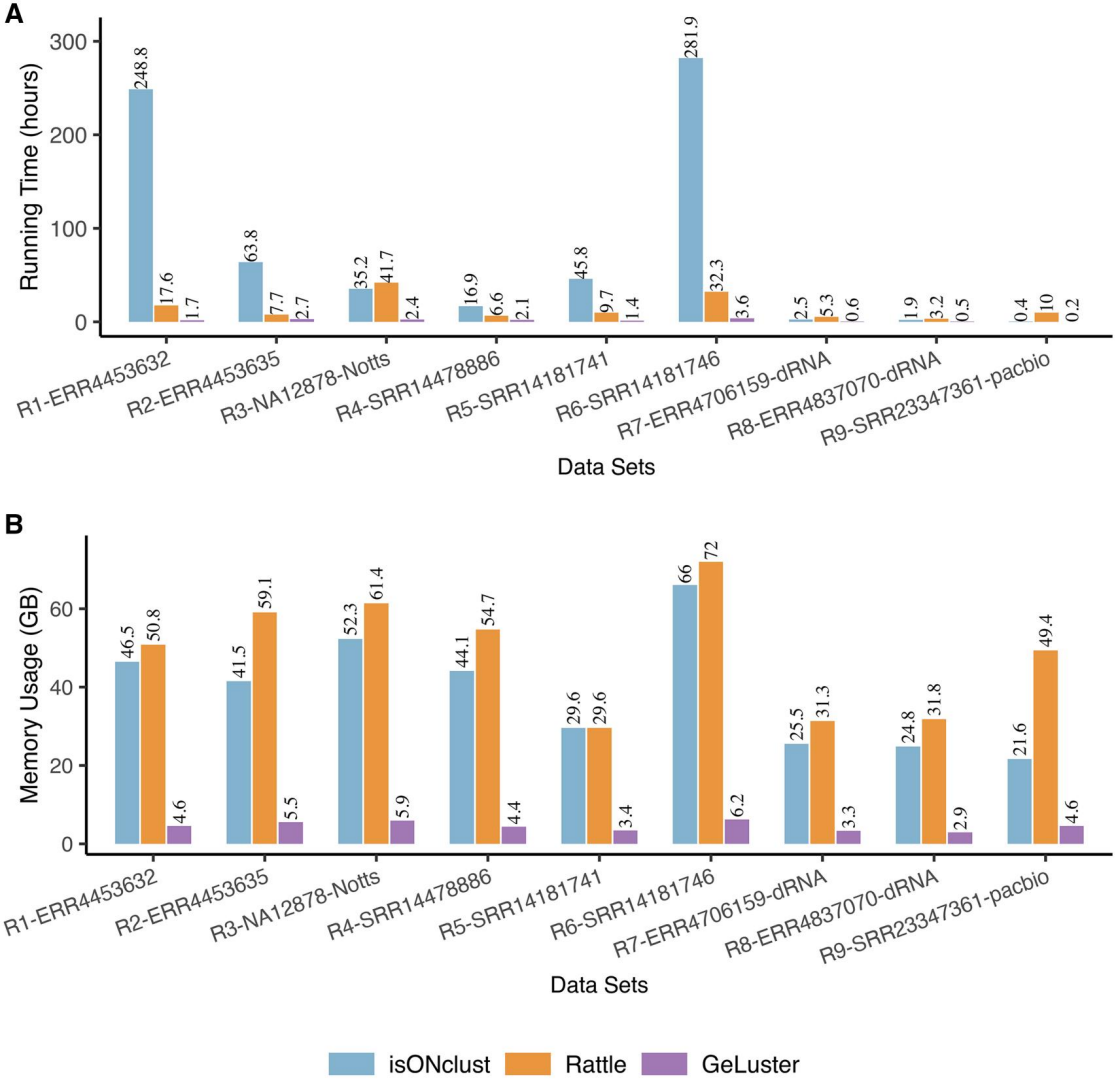
Evaluation of the running time (A) and the memory usage (B) of the clustering tools on the nine real datasets.

De novo processing the sequences generally consume large computing resources (e.g. running time and memory usage). We next investigated the computing resources usage of the clustering tools on the nine real datasets. All the three tools were run on a 2.0 GHz Intel(R) Xeon(R) Platinum server, and RATTLE was run with 24 threads, isONlclust was run with 48 threads, while GeLuster was run with 10 threads on the ONT datasets and 20 threads on the PacBio dataset. As illustrated in Fig. 3A and B, the proposed GeLuster was significantly more efficient compared to the alternatives.

Concretely, the running time of GeLuster on the six cDNA datasets ranged from 1.43  to 3.65 h, which was 2.88–17.5 times as fast as RATTLE, while 7.84–147.79 times faster than isONclust. And, more significantly, GeLuster required much less memory, which was less than one-seventh of the others. Besides, on the two dRNA datasets and the PacBio dataset, GeLuster exhibited more efficient performance as well (see Fig. 3 and Supplementary Table S12 for details). The comparisons of the consumption of the computing resources on the real datasets demonstrated the extremely high efficiency of the proposed GeLuster algorithm, which would lay the foundation for processing large-scale transcriptome sequencing data and for leveraging the greatest achievable benefits of long-read RNA sequencing technology.

#### 3.3.3 Additional tests

We next investigated the clustering accuracy (in terms of ARI and V-Measure) of the tools based on the class size (the number of reads present in a class), which also could be regard as the expression levels of the corresponding genes.

**Figure 4.**
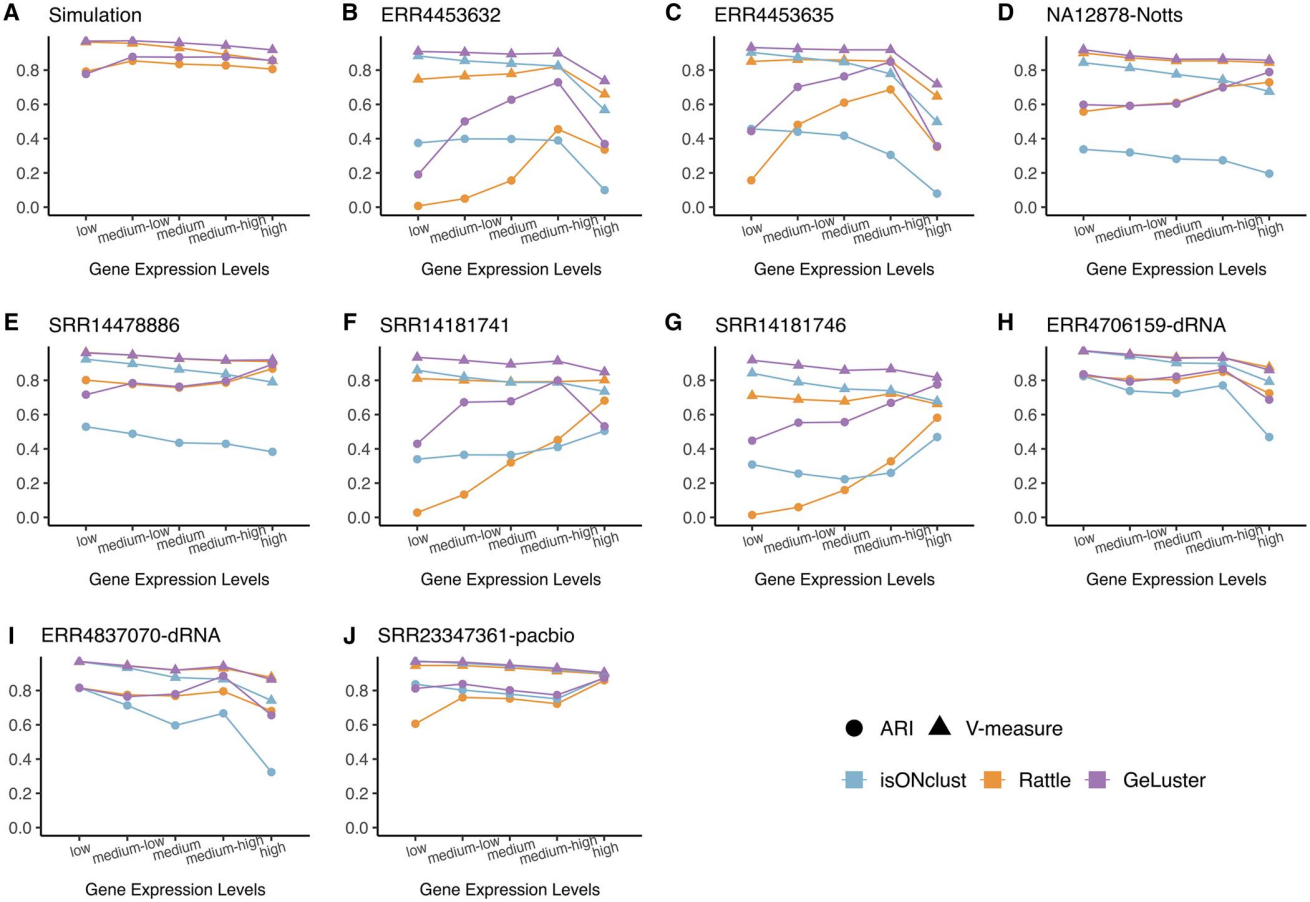
Performance evaluation (in terms of ARI, and V-Measure) of the clustering tools based on the expression abundance of the genes. (A) corresponds to the simulated dataset, (B–G) correspond to the six cDNA datasets, (H) and (I) correspond to the two dRNA datasets, and (J) corresponds to the PacBio dataset.

We first sorted the classes according to the number of reads in them. Then all the classes were equally divided into five parts, which corresponded to low, medium low, medium, medium high, and high expressed ones. Afterwards, we computed the ARI and V-Measure score of the three tools based on each part. The results revealed that GeLuster displayed a better or comparable performance compared with the other methods regardless of the expression levels of the corresponding genes on both the simulated and real datasets (Fig. 4A–I).

Besides, we also compared the performance of the clustering tools in terms of the number of the clusters produced by each tool, the number of reads that were assigned to a cluster. (See Supplementary Tables S2–S11 for more details.)

## 4 Discussion

In this research, we developed a new algorithm to cluster the long-read RNA-seq data into their gene family of origin, namely GeLuster, which is quite different from the traditional approaches. GeLuster avoids doing the time-consuming all-vs-all similarity comparison, as well as avoids the greedy strategy that deeply based on some kind of scoring system between sequences.

From a methodological perspective, GeLuster breaks away from traditional methods. The traditional strategies, no matter the greedy approaches that rely on some kind of scoring system or the all-vs-all pairwise alignment approaches, were to identify similar reads that might originate from the same gene at every stage. On the contrary, GeLuster’s fundamental step focused on identifying sequences that are not similar (i.e. from distinct genes), which is more achievable to a certain extent. This is because to determine whether two sequences are similar, sequence alignment is typically required, but to determine dissimilarity, it is generally sufficient for them to have a small number of shared k-mers. Thus, the first step of GeLuster was to select dissimilar reads from dataset to generate the so-called pseudo-reference, and it is expected that the pseudo-reference contains as much potential genes as possible for the dataset, meanwhile, a gene is expected to correspond to only one subsequence in the pseudo reference. We formulated the problem into a mathematical programming, and designed a heuristic algorithm to resolve it efficiently. After that, through the alignment between reads and the pseudo reference, the reads from the same gene will be clustered naturally. The alignment in the GeLuster package was performed by the well-established and widely used long read alignment tool minimap2, ensuring the high-speed and high-accuracy of the GeLuster.

GeLuster runs in an iterative way, and within each iteration, it extracts a pseudo reference from the reads that need to be clustered, then aligns the reads to the pseudo reference, based on the alignments the reads will be clustered naturally.

Our results demonstrate that GeLuster makes a substantial improvement over existing method. GeLuster outperforms others on almost all the relevant accuracy metrics. More significantly, GeLuster is extremely efficient, which is 2.9–147 times as fast as others with only around one-seventh of memory consumption based on our tests on the ONT data, and two times faster with around one-fifth of memory usage on the PacBio data.

Although we have seen the significant advantages of GeLuster, we still have a long way to go from an application point of view. We would like to combine the proposed GeLuster with a post clustering error-correcting module to reconstruct transcripts de novo from long-read RNA-seq data ultimately. Our priority now is to tackle the error correction problem within each cluster. The ultimate goal is to develop a package for de novo transcript sequence reconstruction.

The software has been developed to be user-friendly and is expected to play a crucial role in transcriptome studies with large-scale long-read RNA-seq data, especially in discovering novel genes in the non-model species. And, we also hope that GeLuster can be used in the study of complicated human diseases related to abnormal splicing events and expression levels, such as cancer, and most genetic diseases.

## Supplementary Material

btae059_Supplementary_Data
