# Supplementary material for: Highly efficient clustering of long-read transcriptomic data with GeLuster
Source: Bioinformatics. 2024 Feb 3;40(2):btae059. doi: 10.1093/bioinformatics/btae059 (PMC10881092; doi:10.1093/bioinformatics/btae059)
Supplement: btae059_Supplementary_Data [file btae059_supplementary_data.docx]

**Supplemental Materials**

**Highly efficient clustering of long-read transcriptomic data with Geluster**

Junchi Ma^1,2^^†^ Xiaoyu Zhao^2†^ Enfeng Qi^3^ Renmin Han^1*^ Ting Yu^1†*^ Guojun Li^1*^

^1^Research Center for Mathematics and Interdisciplinary Sciences, Frontiers Science Center for Nonlinear Expectations (Ministry of Education), Shandong University, Qingdao, 266237, China

^2^School of Mathematics, Shandong University, Jinan, Shandong, 250100, China

^3^School of Mathematics and Statistics, Guangxi Normal University, Guilin 541000, China

^*^ To whom correspondence should be addressed

^†^These authors contributed equally to this work

**1. Supplemental Notes**

**1.1 The version and the parameter setup for the tools used in this research**

The version and running command for the clustering tools on the ONT data are as follows.

| **GeLuster** | v.1 | GeLuster **-r** *reads* **-f** *fq* **-s** *cDNA (or dRNA)* **-o** *geluster_out* **-t** *10* |
| --- | --- | --- |
| **RATTLE** | / | rattle **cluster** **-i** *reads* **-t** 24 **-o** *rattle*_*outdir* |
| **isONclust** | 0.0.6.1 | isONclust **--t** 48 **--ont** **--fastq** *reads* -**-outfolder** i*sonclust_outdir* |
| **CARNAC-LR** | / | minimap2 *reads* *reads* **-X** > *carnac-minimap.paf*  paf_to_CARNAC.py *carnac-minimap.paf* *reads* *input_carnac.txt*  CARNAC-LR **-f** *input_carnac.txt* **-o** *carnac_cluster.txt* |

The version and running command for the clustering tools on the Pacbio data are as follows.

| **GeLuster** | v.1 | GeLuster **-r** *reads* **-f** *fq* -s PacBio **-o** *geluster_out* **-t** *20* |
| --- | --- | --- |
| **RATTLE** | / | rattle **cluster** **-i** *reads* **-t** 24 **-o** *rattle*_*outdir* |
| **isONclust** | 0.0.6.1 | isONclust **--t** 48 **--isoseq** **--fastq** *reads* -**-outfolder** i*sonclust_outdir* |

In this research, we used minimap2 to align the real sequencing reads to the reference genome to generate the ground truth for the real data sets. For accurately aligning, we run minimap2 with the option –junc-bed, which provided the gene annotations in the BED12 format. The version of the reference genome we used was GRCh38 and the gene annotations was hg38.ncbiRefSeq, and both were downloaded from the UCSC Genome Browser (<https://hgdownload.soe.ucsc.edu/>). The version and running command for minimap2 was as follows.

| **minimap2** | 2.24-r1122 | minimap2 **-ax** *splice* **--junc-bed** *bed_file* *ref* *query* >output.sam |
| --- | --- | --- |

**1.2 The long-read RNA-seq data used in this research**

The scripts for data simulation we used in this research were downloaded from <https://github.com/andrewprzh/lrgasp-simulation>. Raw sequence data for the real data sets R1-R2 and R4-R9 can be obtained from NCBI Sequence Read Archive (SRA) under the following accession codes：ERR4453632 (R1), ERR4453635 (R2), SRR14478886 (R4), SRR14181741 (R5), SRR14181746 (R6), ERR4706159 (R7), ERR4837070 (R8), and SRR23347361(R9). And the sequence data for R3 are from the NA12878 RNA sequencing consortium, which can be downloaded from <https://github.com/nanopore-wgs-consortium/NA12878/blob/master/nanopore-human-transcriptome/fastq_fast5_bulk.md>, and what we used was the run2 data set of the 1D cDNA sample sequenced by the University of Nottingham.

**1.3** **The definitions of the accuracy metrics used in this research**

Six accuracy metrices are used to evaluate the performance of the clustering tools in this research, which are H-Score (Homogeneity), C-Score (Completeness), V-Measure (calculated by H-Score and C-Score), ARI, FMI, and NMI. These metrics are detailed described as follows.

**H-Score**: The H-Score measures the homogeneity of a clustering result, with a range of 0 to 1 and 1 indicating perfect homogeneity. It is calculated as the ratio of the number of samples that belong to the most frequent cluster to the total number of samples. The formula for H-Score is as follows:

where C is the true label and K is the predicted label. H(C) is the entropy of C, and H(C|K) is the conditional entropy of C given K.

**C-Score**: The C-Score measures the completeness of a clustering result, with a range of 0 to 1 and 1 indicating perfect completeness. It is calculated as the ratio of the number of samples that belong to the same cluster in the true labels to the total number of samples. The formula for C-Score is similar to that of H-Score.

**V-Measure**: The V-Measure combines both homogeneity and completeness into a single score, with a range of 0 to 1 and 1 indicating perfect clustering. It is calculated as the harmonic mean of homogeneity and completeness. The formula for V-Measure is as follows:

where $\beta$ is weight, h is H-score, c is C-score.

**ARI**: The Adjusted Rand Index (ARI) is a clustering evaluation metric that measures the similarity between two clustering results. It ranges from -1 to 1, with 1 indicating perfect similarity. The ARI is calculated as the difference between the expected index and the index obtained by chance, divided by the maximum possible difference. The formula for ARI is as follows:

where TP is true positive, TN is true negative, FP is false positive, and FN is false negative. RI is the Rand Index, and E(RI) is the expected value of RI.

**FMI**: The Fowlkes-Mallows Index (FMI) is a clustering evaluation metric that measures the similarity between two clustering results. It ranges from 0 to 1, with 1 indicating perfect similarity. The FMI is calculated as the geometric mean of precision and recall. The formula for FMI is similar to that of RI.

**NMI**: The Normalized Mutual Information (NMI) is a clustering evaluation metric that measures the mutual information between two clustering results. It ranges from 0 to 1, with 1 indicating perfect clustering. The NMI is calculated as the mutual information between the true labels and the predicted labels, normalized by the entropy of the true labels and the predicted labels. The formula for NMI is as follows:

where H(X) and H(Y) are the entropy of X and Y, respectively. p(x,y) is the joint probability between x and y, and p(x) and p(y) are the marginal probabilities of x and y. I (X; Y) is the mutual information between X and Y.

All the aforementioned metrics used in this research are calculated using the package sklearn of Python.

**2. Supplemental Tables**

**Table S1.** The detailed description for the constructed ground truth for the real data set. The abbreviation NS is for nonsingleton classes (classes that contain more than one read).

| Data sets | No. of NS | No. of reads | No. of aligned reads | No. of reads in NS |
| --- | --- | --- | --- | --- |
| R1(ERR4453632) | 77,396 | 3,640,754 | 3,347,775 | 3,306,208 |
| R2 (ERR4453635) | 32,838 | 3,639,516 | 3,349,248 | 3,334,243 |
| R3 (NA12878-Notts) | 22,679 | 3,507,034 | 3,436,500 | 3,426,046 |
| R4 (SRR14478886) | 13,683 | 2,365,302 | 2,356,869 | 2,353,257 |
| R5 (SRR14181741) | 20,200 | 1,705,184 | 1,575,018 | 1,566,587 |
| R6 (SRR14181746) | 22,382 | 4,426,087 | 3,833,216 | 3,822,695 |
| R7 (ERR4706159) | 15,395 | 1,815,583 | 1,642,530 | 1,638,236 |
| R8 (ERR4837070) | 15,666 | 1,890,698 | 1,793,354 | 1,788,496 |
| R9 (SRR23347361) | 11,702 | 1,662,081 | 1,656,768 | 1,649,876 |

**Table S2**. The h-core, c-score, V-Measure, ARI, FMI, NMI, the number of nonsingleton classes (classes that contain more than one read), and the number of reads in nonsingleton classes of the tools on the simulated data set. The abbreviation RAT is for RATTLE, ISO for isONclust, GEL for GeLuster, VM for V-Measure, and NS for nonsingleton classes.

| Tools | h-score | c-score | VM | ARI | FMI | NMI | No. of NS | reads in NS |
| --- | --- | --- | --- | --- | --- | --- | --- | --- |
| RAT | 0.950 | 0.916 | 0.933 | 0.791 | 0.793 | 0.933 | 22,932 | 1,474,993 |
| ISO | - | - | - | - | - | - | - | - |
| GEL | 0.968 | 0.950 | 0.959 | 0.852 | 0.853 | 0.959 | 15,411 | 1,409,112 |

**Table S3**. The h-core, c-score, V-Measure, ARI, FMI, NMI, the number of nonsingleton classes (classes that contain more than one read), and the number of reads in nonsingleton classes of the tools on the real data set R1. The abbreviation RAT is for RATTLE, ISO for isONclust, GEL for GeLuster, VM for V-Measure, and NS for nonsingleton classes.

| Tools | h-score | c-score | VM | ARI | FMI | NMI | No. of NS | reads in NS |
| --- | --- | --- | --- | --- | --- | --- | --- | --- |
| RAT | 0.706 | 0.814 | 0.756 | 0.221 | 0.238 | 0.758 | 33,832 | 3,620,288 |
| ISO | 0.917 | 0.765 | 0.834 | 0.138 | 0.228 | 0.838 | 95,582 | 3,531,796 |
| GEL | 0.898 | 0.880 | 0.889 | 0.417 | 0.422 | 0.889 | 42,955 | 3,433,574 |

**Table S4**. The h-core, c-score, V-Measure, ARI, FMI, NMI, the number of nonsingleton classes (classes that contain more than one read), and the number of reads in nonsingleton classes of the tools on the real data set R2. The abbreviation RAT is for RATTLE, ISO for isONclust, GEL for GeLuster, VM for V-Measure, and NS for nonsingleton classes.

| Tools | h-score | c-score | VM | ARI | FMI | NMI | No. of NS | reads in NS |
| --- | --- | --- | --- | --- | --- | --- | --- | --- |
| RAT | 0.802 | 0.849 | 0.825 | 0.390 | 0.403 | 0.825 | 20,307 | 3,628,008 |
| ISO | 0.932 | 0.762 | 0.838 | 0.132 | 0.234 | 0.843 | 48,423 | 3,596,604 |
| GEL | 0.910 | 0.893 | 0.902 | 0.446 | 0.486 | 0.902 | 22,330 | 3,577,943 |

**Table S5**. The h-core, c-score, V-Measure, ARI, FMI, NMI, the number of nonsingleton classes (classes that contain more than one read), and the number of reads in nonsingleton classes of the tools on the real data set R3. The abbreviation RAT is for RATTLE, ISO for isONclust, GEL for GeLuster, VM for V-Measure, and NS for nonsingleton class.

| Tools | h-score | c-score | VM | ARI | FMI | NMI | No. of NS | reads in NS |
| --- | --- | --- | --- | --- | --- | --- | --- | --- |
| RAT | 0.907 | 0.835 | 0.870 | 0.627 | 0.629 | 0.870 | 56,299 | 3,445,588 |
| ISO | 0.907 | 0.712 | 0.798 | 0.223 | 0.316 | 0.804 | 71,179 | 3,416,250 |
| GEL | 0.926 | 0.846 | 0.885 | 0.685 | 0.688 | 0.885 | 35,529 | 3,284,935 |

**Table S6**. The h-core, c-score, V-Measure, ARI, FMI, NMI, the number of nonsingleton classes (classes that contain more than one read), and the number of reads in nonsingleton classes of the tools on the real data set R4. The abbreviation RAT is for RATTLE, ISO for isONclust, GEL for GeLuster, VM for V-Measure, and NS for nonsingleton class.

| Tools | h-score | c-score | VM | ARI | FMI | NMI | No. of NS | reads in NS |
| --- | --- | --- | --- | --- | --- | --- | --- | --- |
| RAT | 0.950 | 0.920 | 0.935 | 0.785 | 0.785 | 0.935 | 17,787 | 2,352,993 |
| ISO | 0.969 | 0.818 | 0.887 | 0.398 | 0.477 | 0.890 | 34,351 | 2,277,937 |
| GEL | 0.958 | 0.918 | 0.938 | 0.818 | 0.819 | 0.938 | 15,701 | 2,330,101 |

**Table S7**. The h-core, c-score, V-Measure, ARI, FMI, NMI, the number of nonsingleton classes (classes that contain more than one read), and the number of reads in nonsingleton classes of the tools on the real data set R5. The abbreviation RAT is for RATTLE, ISO for isONclust, GEL for GeLuster, VM for V-Measure, and NS for nonsingleton class.

| Tools | h-score | c-score | VM | ARI | FMI | NMI | No. of NS | reads in NS |
| --- | --- | --- | --- | --- | --- | --- | --- | --- |
| RAT | 0.736 | 0.832 | 0.781 | 0.233 | 0.308 | 0.782 | 20,528 | 1,678,746 |
| ISO | 0.891 | 0.753 | 0.816 | 0.465 | 0.509 | 0.819 | 38,757 | 1,483,937 |
| GEL | 0.948 | 0.879 | 0.912 | 0.572 | 0.595 | 0.913 | 24,577 | 1,557,350 |

**Table S8**. The h-core, c-score, V-Measure, ARI, FMI, NMI, the number of nonsingleton classes (classes that contain more than one read), and the number of reads in nonsingleton classes of the tools on the real data set R6. The abbreviation RAT is for RATTLE, ISO for isONclust, GEL for GeLuster, VM for V-Measure, and NS for nonsingleton class.

| Tools | h-score | c-score | VM | ARI | FMI | NMI | No. of NS | reads in NS |
| --- | --- | --- | --- | --- | --- | --- | --- | --- |
| RAT | 0.630 | 0.739 | 0.680 | 0.153 | 0.245 | 0.682 | 26,801 | 4,369,955 |
| ISO | 0.905 | 0.700 | 0.789 | 0.421 | 0.492 | 0.796 | 79,372 | 4,113,299 |
| GEL | 0.950 | 0.835 | 0.889 | 0.725 | 0.736 | 0.891 | 32,600 | 3,802,239 |

**Table S9**. The h-core, c-score, V-Measure, ARI, FMI, NMI, the number of nonsingleton classes (classes that contain more than one read), and the number of reads in nonsingleton classes of the tools on the real data set R7. The abbreviation RAT is for RATTLE, ISO for isONclust, GEL for GeLuster, VM for V-Measure, and NS for nonsingleton class.

| Tools | h-score | c-score | VM | ARI | FMI | NMI | No. of NS | reads in NS |
| --- | --- | --- | --- | --- | --- | --- | --- | --- |
| RAT | 0.944 | 0.933 | 0.938 | 0.716 | 0.718 | 0.938 | 16,712 | 1,734,107 |
| ISO | 0.962 | 0.891 | 0.925 | 0.571 | 0.605 | 0.926 | 17,439 | 1,665,820 |
| GEL | 0.960 | 0.924 | 0.942 | 0.718 | 0.727 | 0.942 | 17,670 | 1,702,654 |

**Table S10**. The h-core, c-score, V-Measure, ARI, FMI, NMI, the number of nonsingleton classes (classes that contain more than one read), and the number of reads in nonsingleton classes of the tools on the real data set R8. The abbreviation RAT is for RATTLE, ISO for isONclust, GEL for GeLuster, VM for V-Measure, and NS for nonsingleton class.

| Tools | h-score | c-score | VM | ARI | FMI | NMI | No. of NS | reads in NS |
| --- | --- | --- | --- | --- | --- | --- | --- | --- |
| RAT | 0.938 | 0.924 | 0.931 | 0.650 | 0.653 | 0.931 | 15,190 | 1,869,500 |
| ISO | 0.953 | 0.862 | 0.905 | 0.440 | 0.493 | 0.906 | 17,750 | 1,871,988 |
| GEL | 0.959 | 0.917 | 0.937 | 0.696 | 0.708 | 0.937 | 17,647 | 1,856,013 |

**Table S11**. The h-core, c-score, V-Measure, ARI, FMI, NMI, the number of nonsingleton classes (classes that contain more than one read), and the number of reads in nonsingleton classes of the tools on the real data set R9. The abbreviation RAT is for RATTLE, ISO for isONclust, GEL for GeLuster, VM for V-Measure, and NS for nonsingleton class.

| Tools | h-score | c-score | VM | ARI | FMI | NMI | No. of NS | reads in NS |
| --- | --- | --- | --- | --- | --- | --- | --- | --- |
| RAT | 0.928 | 0.920 | 0.924 | 0.768 | 0.769 | 0.924 | 15,800 | 1,651,293 |
| ISO | 0.960 | 0.925 | 0.942 | 0.812 | 0.814 | 0.942 | 18,970 | 1,635,491 |
| GEL | 0.966 | 0.933 | 0.949 | 0.835 | 0.836 | 0.949 | 17,596 | 1,640,877 |

**Table S12** Comparison of running time and memory usage for the clustering tools on the nine real data sets**.**

|  |  | RATTLE | isONclust | GeLuster |
| --- | --- | --- | --- | --- |
| R1 ERR4453632 | running time(hours) | 17.63 | 248.78 | 1.68 |
|  | Maximum memory (GB) | 50.85 | 46.54 | 4.60 |
| R2 ERR4453635 | running time(hours) | 7.68 | 63.83 | 2.67 |
|  | Maximum memory (GB) | 59.11 | 41.47 | 5.53 |
| R3  NA12878-Notts | running time(hours) | 41.70 | 35.22 | 2.42 |
|  | Maximum memory (GB) | 61.36 | 52.34 | 5.88 |
| R4 SRR14478886 | running time(hours) | 6.55 | 16.85 | 2.15 |
|  | Maximum memory (GB) | 54.69 | 44.08 | 4.42 |
| R5 SRR14181741 | running time(hours) | 9.70 | 45.80 | 1.43 |
|  | Maximum memory (GB) | 29.56 | 29.62 | 3.41 |
| R6 SRR14181746 | running time(hours) | 32.28 | 281.85 | 3.65 |
|  | Maximum memory (GB) | 71.99 | 66.03 | 6.18 |
| R7 ERR4706159 | running time(hours) | 5.32 | 2.45 | 0.63 |
|  | Maximum memory (GB) | 31.33 | 25.53 | 3.28 |
| R8 ERR4837070 | running time(hours) | 3.22 | 1.92 | 0.53 |
|  | Maximum memory (GB) | 31.76 | 24.75 | 2.94 |
| R9  SRR23347361 | running time(hours) | 9.97 | 0.41 | 0.20 |
|  | Maximum memory (GB) | 49.35 | 21.62 | 4.62 |
